# Polyendocrine–Metabolic Profile in Adolescents and Young Women with Ovulatory Dysfunction: A Cross-Sectional Study

**DOI:** 10.3390/biology15131041

**Published:** 2026-06-30

**Authors:** Juan Pablo Del Río, Hugo Soto, Patricio Contreras, Álvaro Becerra, Camila Contreras-Rojo, Manuel E. Cortés, Pilar Vigil

**Affiliations:** 1 Departamento de Psiquiatría y Salud Mental, Facultad de Medicina, Universidad de Chile, Santiago 8380453, Chile; jdelrio@ug.uchile.cl; 2 Reproductive Health Research Institute (RHRI), Santiago 8330078, Chile; hsoto@rhri.cl (H.S.); pathomero@gmail.com (P.C.); camilacontrerasrojo@gmail.com (C.C.-R.); 3 Escuela de Fonoaudiología y Departamento de Ciencias Químicas y Biológicas, Universidad Bernardo O’Higgins, Santiago 8370993, Chile; 4 Dirección de Investigación, Universidad Bernardo O’Higgins, Santiago 8370993, Chile; cortesmanuel@docente.ubo.cl

**Keywords:** hyperandrogenemia, hyperprolactinemia, infertility, insulin resistance, ovulatory dysfunction, polycystic ovary syndrome, polyendocrine metabolic ovarian syndrome, thyroid dysfunction

## Abstract

(1) Background: Ovulatory dysfunction (OD) is frequently the first clinical expression of an underlying polyendocrine or metabolic disturbance. In the context of the international consensus renaming polycystic ovary syndrome (PCOS) as polyendocrine metabolic ovarian syndrome (PMOS), our primary aim was to estimate the prevalence of four OD-associated endocrinopathies—hyperandrogenemia, insulin resistance (IR), thyroid dysfunction, and hyperprolactinemia—in a Chilean symptomatic clinical cohort, and to compare their frequencies between adolescents and young adults. (2) Methods: Cross-sectional study including 251 women (≥2 years post-menarche) with confirmed OD, stratified into Group A (12–18 years; *n* = 96) and Group B (19–35 years; *n* = 155). (3) Results: Hyperandrogenemia was the most prevalent endocrinopathy in both groups (50.0% vs. 49.0%; *p* = 0.882), driven primarily by elevated dehydroepiandrosterone sulfate (DHEAS) in Group A (37.5% vs. 24.5%; *p* = 0.028). IR was significantly more frequent in adolescents (55.2% vs. 41.3%; *p* = 0.032). Thyroid dysfunction (primary threshold: thyroid-stimulating hormone [TSH] > 5.0 μIU/mL) was present in 2.1% and 5.2% of Groups A and B (*p* = 0.226). Hyperprolactinemia (>25 ng/mL; macroprolactin-excluded) was detected in 13.5% and 13.6% (*p* = 0.999). (4) Conclusions: All four endocrinopathies were detectable during adolescence, with prevalences largely comparable to those observed in young adult women, supporting the rationale for considering structured polyendocrine evaluation in women with OD ≥ 2 years post-menarche within the PAInT (Prolactin, Androgens, Insulin, and Thyroid hormones) framework.

## Simple Summary

Irregular menstrual cycles are common in adolescents and young women and are frequently the first sign of an underlying hormonal imbalance. In this study, 251 Chilean women aged 12 to 35 years who presented with ovulatory dysfunction at a reproductive health clinic in Santiago were evaluated for four hormonal conditions: excess androgens (hyperandrogenemia), impaired ability to regulate blood glucose (insulin resistance), thyroid gland abnormalities, and elevated prolactin. All four conditions were already present during adolescence at frequencies comparable to those in young adult women. Excess androgens affected approximately half of all participants, while insulin resistance was more frequent in adolescents than in adults. Thyroid and prolactin abnormalities occurred at similar frequencies in both age groups. These results support the value of systematic hormonal evaluation in young women with menstrual irregularities from the time ovulation is expected to be physiologically established. Early identification may facilitate appropriate endocrine evaluation and help prevent long-term reproductive and cardiometabolic complications.

## 1. Introduction

Ovulation is a pivotal physiological event and a recognized biomarker of systemic endocrine health [1]. It results from the coordinated activity of the hypothalamic–pituitary–adrenal–gonadal (HPAG) axis, which regulates follicular recruitment, steroidogenesis, and the preovulatory LH surge through tightly controlled positive and negative feedback mechanisms [1,2]. Menarche marks the initiation of gonadotropin-dependent ovarian cyclicity; anovulatory cycles are physiologically expected during the first two years post-menarche owing to incomplete maturation of the kisspeptinergic GnRH pulse generator [3]. Once the reproductive axis matures, healthy women exhibit regular ovulatory cycles of 24–38 days [4].

Ovulatory dysfunction (OD) is defined as abnormal or absent ovulation (≤9 events/year) and is considered present when a woman exhibits more than two consecutive irregular cycles (<24 or >38 days), three or more non-consecutive irregular cycles per year, a persistent luteal phase < 9 days, serum progesterone < 5 ng/mL on day 21, or ultrasound evidence of anovulation [4]. Beyond menstrual irregularity, OD is associated with hirsutism, acne, alopecia, weight gain, and mood disorders, leading patients to consult across multiple specialties [4,5].

The International Federation of Gynecology and Obstetrics (FIGO) Ovulatory Disorders Classification System categorizes OD into hypothalamic, pituitary, ovarian, and polyendocrine–metabolic etiologies [4,6]. This taxonomy acquires renewed significance in the context of the recent international consensus renaming polycystic ovary syndrome (PCOS) as polyendocrine metabolic ovarian syndrome (PMOS) [7]. This change recognizes that the condition is primarily defined by systemic polyendocrine–metabolic dysfunction rather than by ovarian morphology alone [7].

**Figure 1:**
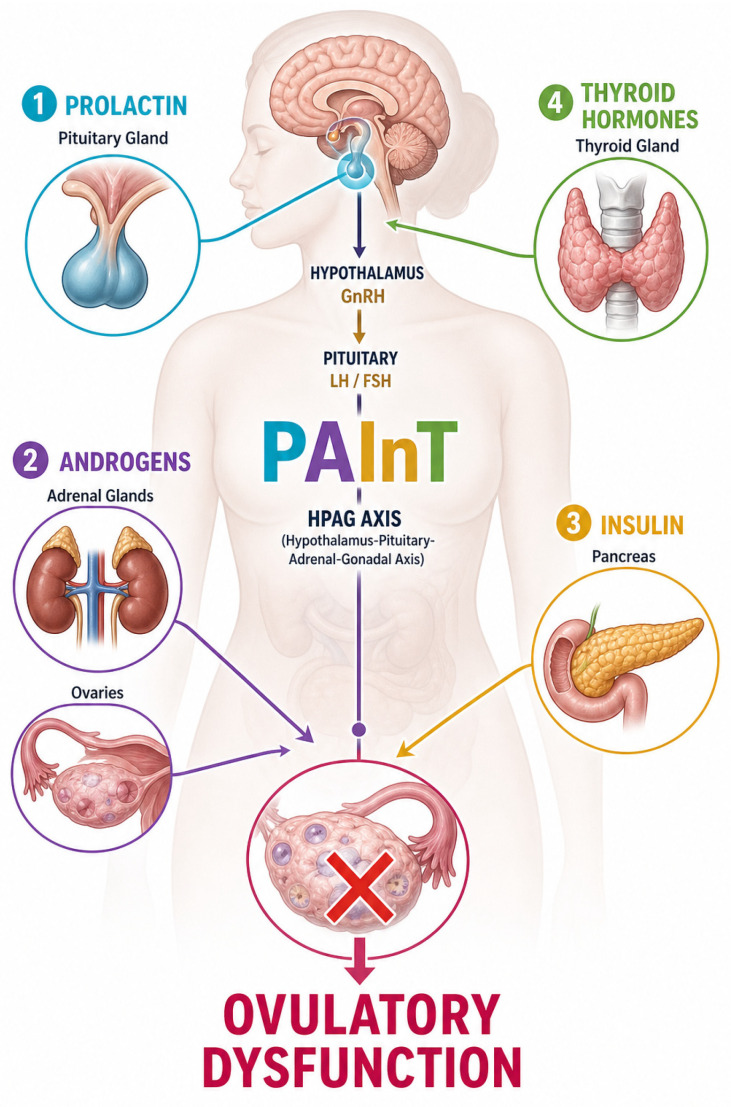
The PAInT framework: principal endocrine etiologies of ovulatory dysfunction. Prolactin (P), androgens (A), insulin (In), and thyroid hormones (T) influence the hypothalamic–pituitary–ovarian axis through neuroendocrine and metabolic mechanisms, including modulation of the kisspeptin/GnRH pathway, thereby contributing to altered folliculogenesis and OD [1,3,7,8,9,10,11].

The kisspeptinergic system integrates reproductive and metabolic signals—including estradiol, prolactin, insulin, leptin, and androgen signaling—and alterations in these regulatory pathways may contribute to OD [3]. The four endocrinopathies most consistently associated with OD are integrated into the PAInT (Prolactin, Androgens, Insulin, and Thyroid hormones) framework [1,3,8,9,10,11], as shown in Figure 1. The PAInT framework provides a structured approach for systematic endocrine evaluation and is consistent with the polyendocrine–metabolic construct underlying the PMOS nomenclature [7].

Despite international guidelines acknowledging the importance of investigating these conditions in women with OD [4,12], systematic endocrine evaluation is frequently omitted in adolescents not seeking pregnancy, partly due to the assumption that irregular cycles reflect physiological HPAG immaturity [13]. Available evidence indicates that hyperandrogenemia, IR, thyroid dysfunction, and hyperprolactinemia are already detectable during adolescence at prevalences comparable to adult populations [14,15], and early identification may facilitate timely intervention and prevent long-term reproductive and cardiometabolic sequelae [15,16].

Whether the four principal OD-associated endocrinopathies are already established during adolescence at frequencies comparable to those in young adult women remains insufficiently characterized in Latin American populations, a gap with direct implications for the timing of clinical evaluation within the PAInT framework. Determining whether these endocrine disturbances are prevalent during adolescence is clinically relevant because it informs the appropriate age at which structured polyendocrine evaluation should be initiated, consistent with the polyendocrine–metabolic construct formalized in the PMOS nomenclature [7]. Our primary aim was to estimate the prevalence of four OD-associated endocrinopathies—hyperandrogenemia, insulin resistance (IR), thyroid dysfunction, and hyperprolactinemia—in a Chilean symptomatic clinical cohort. The secondary aim was to compare their frequencies between adolescents (12–18 years; ≥2 years post-menarche) and young adult women (19–35 years), to determine whether structured PAInT-based endocrine evaluation is warranted from early adolescence onward.

## 2. Materials and Methods

### 2.1. Study Design and Setting

**Figure 2:**
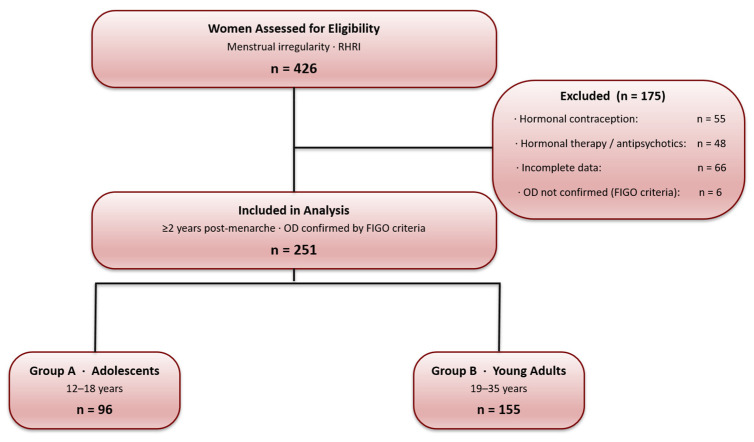
Flow diagram of participant enrollment, inclusion, and analysis according to the STROBE guidelines.

This cross-sectional, single-center observational prevalence study included 251 women aged 12–35 years with confirmed OD attending a gynecological center (Reproductive Health Research Institute, RHRI) located in Santiago, Chile (Figure 2). All participants were ≥2 years post-menarche and were predominantly of self-reported mixed Hispanic/Mestizo ancestry, consistent with the predominant ethnic composition of Santiago’s urban population (INE 2017 Census) [17]. Participants were stratified into Group A (adolescents, 12–18 years; *n* = 96) and Group B (young adults, 19–35 years; *n* = 155). Women using hormonal contraception or receiving pharmacological treatment for a previously diagnosed endocrine condition in the preceding six months were excluded. The study was approved by the RHRI Ethics Committee and conducted in accordance with the Declaration of Helsinki. Written informed consent was obtained from all adult participants; written parental or legal guardian consent was additionally obtained for all minor participants in compliance with Chilean Law 20,584 and Ministry of Health (MINSAL) guidelines.

This manuscript was prepared and reported in accordance with the Strengthening the Reporting of Observational Studies in Epidemiology (STROBE) guidelines for cross-sectional studies [18].

### 2.2. OD Diagnosis

OD was diagnosed in accordance with the FIGO criteria [4]: more than two consecutive irregular cycles (<24 or >38 days), ≥3 non-consecutive irregular cycles per year, a persistent luteal phase < 9 days [4], serum progesterone < 5 ng/mL on day 21, or ultrasound evidence of anovulation. In adolescents, menstrual irregularity and hyperandrogenism were interpreted cautiously per the 2023 International Evidence-based Guideline for PMOS [12], which recommends that adolescents with suggestive but not full diagnostic criteria be considered ‘at increased risk’ and reassessed at reproductive maturity. OD was not diagnosed during breastfeeding, pregnancy, or perimenopause [1,4].

### 2.3. Clinical Evaluation and Basal Hormonal Profile

Each participant underwent a comprehensive clinical evaluation and a complete basal hormonal profile (BHP) comprising FSH, estradiol (E2), thyroid-stimulating hormone (TSH), prolactin (PRL), dehydroepiandrosterone sulfate (DHEAS), androstenedione (A4), total testosterone (TT), sex hormone-binding globulin (SHBG), albumin, calculated free testosterone (CFT), and 17-OH-progesterone (17-OHP). An oral glucose tolerance test with insulin measurements (five-step OGTT-I) was also performed to assess glucose metabolism and insulin dynamics. Blood samples were drawn on days 3–5 of the menstrual cycle (menstruating participants) or on a random day (amenorrhoeic participants), under fasting conditions before 09:00 h. All assays were performed at a single certified clinical laboratory in accordance with ISO 15189 quality assurance protocols.

### 2.4. IR Assessment

The OGTT-I was conducted after a 12 h overnight fast (75 g anhydrous glucose). Blood samples were collected at 0, 30, 60, 90, and 120 min for simultaneous plasma glucose (hexokinase method) and serum insulin (chemiluminescence immunoassay). IR was defined by ≥1 of (i) homeostatic model assessment index (HOMA) > 2.09, corresponding to the 75th percentile of the Chilean adult reference population [19]; (ii) insulin sensitivity index (ISI) composite < 4.45 [19]; or (iii) I_0_ × G_60_ > 1110, validated in Chilean women [19]. The use of three complementary indices captures IR phenotypes that fasting surrogates alone do not detect.

### 2.5. Hyperandrogenemia Assessment

Hyperandrogenemia was defined by ≥1 of the following criteria: TT > 47 ng/dL, CFT > 9 pg/mL, DHEAS > 250 μg/dL, or A4 > 2.8 ng/mL [20]. The diagnostic thresholds used to define hyperandrogenemia (TT > 47 ng/dL, CFT > 9 pg/mL, DHEAS > 250 μg/dL, and A4 > 2.8 ng/mL) were selected on the basis of the following: (i) the upper reference limits established by the assay manufacturer for adult premenopausal women (TOSOH Bioscience and Beckman Coulter certified laboratory reference intervals); (ii) the Androgen Excess and PCOS Society (AE-PCOS) criteria for biochemical hyperandrogenism [20]; and (iii) consistency with cutoffs used in validated reproductive endocrinology cohorts employing LC-MS/MS measurements of androgen excess [14]. It is acknowledged that age- and assay-specific androgen reference intervals are not fully established for adolescent females when using immunoassay platforms, and that adrenal DHEAS rises physiologically during adrenarche, a process that extends into mid-adolescence [13,15]. Accordingly, DHEAS elevations in Group A must be interpreted cautiously, as a proportion of values above the adult threshold may represent the upper end of the normal pubertal adrenarche trajectory rather than pathological hyperandrogenism. Definitive characterization of androgen excess in adolescents will require age- and Tanner stage-specific reference intervals derived from LC-MS/MS measurements, which were not available for this cohort. TT and DHEAS were quantified by ELISA (TOSOH Bioscience, San Francisco, CA, USA), SHBG by immunoradiometric assay (Beckman Coulter, Brea, CA, USA), and CFT was calculated using the Vermeulen mass-action equation [21]. 17-OHP was measured by ELISA to exclude non-classic 21-hydroxylase-deficient congenital adrenal hyperplasia (basal threshold ≥ 2 ng/mL) [22]. Given the known immunoassay limitations at low female androgen concentrations compared with LC-MS/MS, all hyperandrogenemia prevalences should be regarded as upper-bound estimates [14].

### 2.6. Hyperprolactinemia Assessment

Hyperprolactinemia was defined as basal serum PRL > 25 ng/mL [23]. All values >25 ng/mL were re-analyzed using a low macroprolactin-reactivity immunoradiometric method (polyethylene glycol [PEG] precipitation; post-precipitation recovery threshold: ≥40% of initial PRL value retained as monomeric prolactin) to exclude macroprolactin prior to classification [23]. Borderline-elevated values were managed by recommending repeat sampling as previously described [23]. Participants with known PRL-elevating medications or previously diagnosed pituitary adenomas were excluded from hyperprolactinemia prevalence analyses.

### 2.7. Thyroid Function Assessment

Thyroid hormones were measured by direct chemiluminescence immunoassay (Beckman Coulter Access 2; intra-assay CV: 2.1%; inter-assay CV: 4.3%). For the primary analysis, elevated TSH was defined as >5.0 μIU/mL, the certified laboratory upper reference limit [24,25]. An exploratory secondary analysis examined the effect of two additional TSH thresholds on the apparent prevalence of thyroid dysfunction: TSH > 4.5 μIU/mL (ATA/ASRM upper normal limit [24,25]) and TSH > 2.5 μIU/mL (a lower threshold used in some reproductive medicine contexts [24,25]). The category ‘TSH 2.5–5.0 μIU/mL’ is designated an exploratory upper reference range (URR) with no diagnostic or treatment implication under current ATA [24,26], ASRM [25], or European Society of Human Reproduction and Embryology (ESHRE) [27] guidelines for non-pregnant reproductive-age women.

It must be emphasized that women with TSH values in the 2.5–5.0 μIU/mL range (upper reference range, URR) are within the certified laboratory normal reference interval and do not meet diagnostic criteria for hypothyroidism, subclinical hypothyroidism, or any thyroid disorder under current guidelines from the American Thyroid Association (ATA) [24,26], the American Society for Reproductive Medicine (ASRM) [25], or the European Society of Human Reproduction and Embryology (ESHRE) [27] for non-pregnant reproductive-age women. The exploratory TSH > 2.5 μIU/mL analysis is presented solely as a descriptive sensitivity analysis to illustrate threshold-dependence in apparent prevalence estimation; it carries no diagnostic or therapeutic implication, and no clinical action should be inferred from URR status alone.

### 2.8. Premature Ovarian Insufficiency (POI)

POI was diagnosed when early follicular FSH > 25 IU/L was accompanied by serum E2 < 50 pg/mL on two occasions ≥4 weeks apart, as proposed by the ESHRE guidelines [27].

### 2.9. External Reference Populations

In the absence of a concurrent control group with confirmed ovulatory function, prevalence estimates were contextualized against four validated external references. All comparisons are descriptive and are not equivalent to concurrent matched within-study controls: (i) IR: HOMA ≥ 2.6 in 16.3% of 667 healthy Chilean adolescents [28]; (ii) thyroid dysfunction: clinical hypothyroidism in 0.6% (women 15–24 years) and 1.6% (25–44 years) per ENS 2016-2017 (MINSAL) [17]; (iii) hyperprolactinemia: 4.1% (95% CI: 2.1–6.0%) in asymptomatic premenopausal blood donors [29]; and (iv) hyperandrogenism: 6–10% in unselected reproductive-age women [30].

The absence of a concurrent group of healthy, ovulatory women constitutes an important limitation of this study. External reference populations were used to contextualize prevalence estimates, but these comparisons are descriptive only; differences in recruitment setting, ethnicity, assay methodology, and calendar period preclude direct equivalence to matched within-study controls. Accordingly, the prevalence estimates reported herein reflect a symptomatic care-seeking cohort and should not be interpreted as demonstrating excess endocrinopathy prevalence relative to ovulatory women without formal inferential testing against a concurrent control group.

### 2.10. Statistical Analysis

Data normality was assessed by the Shapiro–Wilk test. Continuous variables are reported as the mean (± SD) or median (IQR); categorical variables as *n* (%). Between-group comparisons used Student’s *t* test or Mann–Whitney U test for continuous variables and Fisher’s exact test for categorical variables (two-tailed). A post hoc power assessment for the primary between-group IR comparison (55.2% vs. 41.3%; Group A *n* = 96, Group B *n* = 155) confirmed 73.2% statistical power at α = 0.05 (G*Power v3.1). All analyses were performed in Stata v12.0 (StataCorp, College Station, TX, USA); *p* < 0.05 was considered statistically significant.

All analyses were conducted using a descriptive and exploratory framework; no multivariable modelling or formal adjustment for multiple comparisons was performed, and *p*-values should therefore be interpreted cautiously in this context.

## 3. Results

### 3.1. Sample Characteristics

A total of 251 women met the inclusion criteria: Group A (*n* = 96; 38.3%) and Group B (*n* = 155; 61.7%). Significant differences were observed between groups for age (*p* < 0.0001) and weight (*p* = 0.020), whereas height and nutritional status did not differ significantly. The prevalence of excess weight (overweight or obesity) was comparable between groups (37.5% vs. 35.5%; *p* = 0.747). Full demographic and anthropometric characteristics are presented in Table 1.

### 3.2. Main Reason for Consultation

Presenting symptoms are summarized in Table 2. Irregular menses was the primary reason for consultation in both groups (58.3% vs. 48.4%; *p* = 0.138). Acne was significantly more frequent in Group A (40.6% vs. 18.7%; *p* < 0.001). Infertility was reported exclusively in Group B (16.1%; *p* < 0.001). More than one symptom could coexist within the same participant.

### 3.3. Basic Hormonal Profile

Table 3 presents the complete hormonal and metabolic parameters. Group B showed significantly higher FSH (median 6.45 vs. 5.11 IU/L; *p* < 0.0001) and estradiol (55.00 vs. 36.18 pg/mL; *p* = 0.005). DHEAS was significantly higher in Group A (218.00 vs. 191.00 μg/dL; *p* = 0.022). Group A exhibited significantly higher fasting insulin (10.30 vs. 7.35 μIU/mL; *p* = 0.002) and HOMA index (2.16 vs. 1.59; *p* = 0.003). TSH, PRL, TT, CFT, SHBG, A4, and fasting glycaemia did not differ significantly between groups.

### 3.4. Prevalence of Endocrinopathies

The prevalence of each endocrinopathy, with per-criterion breakdown, is shown in Table 4. Hyperandrogenemia was the most prevalent endocrinopathy in both groups (50.0% vs. 49.0%; *p* = 0.882), driven primarily by elevated DHEAS in Group A (37.5% vs. 24.5%; *p* = 0.028). IR was significantly more frequent in adolescents (55.2% vs. 41.3%; *p* = 0.032), with convergent evidence from HOMA > 2.09 (47.9% vs. 27.1%; *p* = 0.001) and ISI composite < 4.45 (46.9% vs. 33.6%; *p* = 0.035). The I0 × G60 > 1110 criterion did not differ significantly between groups (36.5% vs. 27.7%; *p* = 0.147). Using the primary diagnostic threshold of TSH > 5.0 μIU/mL, thyroid dysfunction was present in 2.1% and 5.2% of Groups A and B, respectively (*p* = 0.226). An exploratory secondary analysis applying TSH > 2.5 μIU/mL found that 28.1% and 28.4% exceeded this cutoff (*p* = 0.964); this approximately 11-fold amplification is driven entirely by women with TSH in the upper reference range (URR: 2.5–5.0 μIU/mL), who remain within the laboratory normal reference interval and do not qualify for hypothyroidism diagnosis under ATA [24,26] or ASRM [25] guidelines. Accordingly, all primary prevalence claims use the TSH > 5.0 μIU/mL threshold. Hyperprolactinemia (macroprolactin-excluded) was comparable between groups (13.5% vs. 13.6%; *p* = 0.999). POI was identified in two participants in Group B only (1.3%; *p* = 0.264).

## 4. Discussion

### 4.1. Polyendocrine–Metabolic Profile of Ovulatory Dysfunction

This study characterizes the polyendocrine–metabolic profile of Chilean adolescents and young adult women with confirmed OD. All four endocrinopathies—hyperandrogenemia, IR, thyroid dysfunction, and hyperprolactinemia—were detectable during adolescence at prevalences statistically comparable to those in young adults, supporting the rationale for early structured evaluation within the PAInT framework [8,9,10,11,23,24,25,26]. These findings align with the polyendocrine–metabolic construct formalized in the PMOS nomenclature [7], in which systemic endocrine disturbances across the androgen, insulin, thyroid, and prolactin axes co-exist within a shared pathophysiological substrate [20,23,24,25,26].

The prevalence of menstrual disorders in the present cohort (58.3% in Group A, 48.4% in Group B presenting with irregular menses) is substantially higher than population-based estimates of 14.2–27% [31,32]. This discrepancy likely reflects selection bias inherent to a clinical cohort and should not be extrapolated to community-level prevalence. Importantly, all participants were ≥2 years post-menarche, which substantially reduces the likelihood that cycle irregularity solely reflects physiological HPAG immaturity, given that the kisspeptinergic GnRH pulse generator reaches functional maturity within this developmental window [3,13]. When persistent cycle irregularity occurs beyond this developmental period, a pathological etiology warrants active investigation [4,13].

The four endocrinopathies most consistently associated with OD are integrated in the PAInT framework [8,9,10,11,23,24,25,26], which provides a structured four-axis clinical protocol for systematic endocrine evaluation:

#### 4.1.1. Hyperandrogenemia

Approximately half of all participants exhibited biochemical hyperandrogenemia, making it the most prevalent endocrinopathy in this cohort. This observation is consistent with evidence linking androgen excess to ovulatory dys-function through altered ovarian insulin signaling and glucose transport [11], the rec-ognized association between hyperandrogenism, chronic anovulation and PCOS-related menstrual irregularity [20], kisspeptin-related dysregulation of GnRH/gonadotrophin secretion [33], reduced hepatic SHBG production with consequent increased androgen bioavailability [34,35], the pubertal decline in insulin sensitivity [36,37], androgenic suppression in insulin-sensitive and insulin-resistant PCOS phenotypes [38], molecular disturbances in insulin signaling and carbohydrate metabolism in PCOS [39], the adverse impact of insulin resistance on fertility [40], and the association of hyperinsulinemia and dysglycemia with the severity of menstrual dysfunction [41]. The adrenal component was more prominent in adolescents, evidenced by significantly higher DHEAS in Group A (37.5% vs. 24.5%; *p* = 0.028), consistent with the adrenarche-dominated androgenic milieu of this developmental stage [13,15]. Non-classic 21-hydroxylase-deficient congenital adrenal hyperplasia was excluded in all participants by basal 17-OHP measurement [22]. The observed prevalence of hyperandrogenemia in this symptomatic cohort (~50%) considerably exceeds the 6–10% prevalence of PCOS reported in unselected reproductive-age women [6,30], an excess compatible with the care-seeking nature of this population. All hyperandrogenemia prevalence estimates should be interpreted as upper-bound values given the limitations of immunoassay relative to LC-MS/MS for female androgen quantification [14]. In addition, the definition of hyperandrogenemia in adolescents warrants particular caution because age and assay-specific androgen reference intervals are not fully established for this population when using immunoassay methodology [13,14,20]. Physiological adrenarche extends into mid-adolescence, and DHEAS values above adult thresholds in Group A may therefore represent the upper end of normal pubertal development rather than unequivocal pathological androgen excess [13,15]. Consequently, hyperandrogenemia prevalences in adolescents should be interpreted as upper-bound estimates pending the availability of Tanner stage-specific reference ranges derived from LC-MS/MS measurements [14]. All androgen measurements were performed by immunoassay (ELISA for TT and DHEAS; immunoradiometric assay for SHBG), with CFT calculated using the Vermeulen equation. Immunoassays are subject to well-documented limitations in the female androgen range, including cross-reactivity with steroid precursors and binding proteins, imprecision at low concentrations, and matrix-dependent interference [14]. These methodological constraints are relevant to the interpretation of TT prevalence in particular, as TT values in women frequently fall near or below the functional sensitivity threshold of many immunoassay platforms. Liquid chromatography-tandem mass spectrometry (LC-MS/MS) is the reference method for androgen quantification in women and consistently yields lower androgen concentrations than immunoassay under equivalent conditions [14]. The current findings therefore represent upper-bound estimates of hyperandrogenemia prevalence, and validation of the observed rates using LC-MS/MS in a subset of the cohort is a priority for future work.

#### 4.1.2. Insulin Resistance

Physiological insulin resistance during puberty is a well-characterized phenomenon [35,36,37], peaking at Tanner stage 3–4 and normalizing following the completion of Tanner stage 5 [36,37]. Its mechanism involves growth hormone-mediated post-receptor insulin signaling impairment and is independent of adiposity [36,37,39]. In the present cohort, pubertal staging (Tanner stage) was not systematically documented, a recognized limitation that prevents formal partitioning of the observed IR excess in Group A into its physiological and pathological components. However, several lines of evidence support the inference that at least a portion of the observed IR excess in adolescents reflects pathological metabolic dysregulation rather than pubertal physiology alone [35,39,40,41]: (i) all participants were ≥2 years post-menarche, a developmental milestone that in most girls corresponds to Tanner stage 4–5 completion and normalization of pubertal IR [13]; (ii) IR prevalence in Group A (55.2%) was approximately 3.4-fold above the reference prevalence of HOMA ≥ 2.6 in healthy Chilean adolescents matched for age and BMI distribution [28]; and (iii) the excess was detected convergently by three independent criteria (HOMA, ISI composite, and I_0_ × G_60_), reducing the likelihood that it reflects measurement artefacts or threshold misclassification alone [19]. Nevertheless, residual confounding by pubertal physiological IR cannot be excluded with certainty in the absence of Tanner staging data, and this represents a limitation that future studies in this population should address by incorporating formal pubertal staging at enrolment.

#### 4.1.3. Thyroid Dysfunction

Elevated or high-normal TSH values have been associated with female subfertility, reduced fecundity, or unexplained infertility in observational reproductive medicine studies [42,43,44]. Using the primary diagnostic threshold of TSH > 5.0 μIU/mL, thyroid dysfunction was present in 2.1% of adolescents and 5.2% of young adults, values numerically higher than those reported in the Chilean National Health Survey but within the same order of magnitude [17]. An exploratory secondary analysis applying a lower threshold of TSH ≥ 2.5 μIU/mL yielded an apparent thyroid “dysfunction” prevalence of 28.1–28.4%, representing an approximately 11-fold amplification driven entirely by women with TSH values in the upper reference range (2.5–5.0 μIU/mL) [45,46]. These values remain within the certified laboratory reference interval and do not meet the diagnostic criteria for hypothyroidism under current ATA and ASRM guidelines for non-pregnant reproductive-age women [24,25,26]. Accordingly, this exploratory analysis is reported to illustrate the magnitude of threshold-dependent variability in prevalence estimates rather than to support lowering diagnostic or treatment thresholds, for which evidence remains inconsistent [47] all primary prevalence claims and interpretative statements in this study therefore rely on the TSH > 5.0 μIU/mL threshold.

#### 4.1.4. Hyperprolactinemia

Hyperprolactinemia prevalence was 13.5% (Group A) and 13.6% (Group B), approximately 3.3-fold above the 4.1% reported in asymptomatic premenopausal blood donors [29] and higher than the 1.7–5.5% reported in adolescents with menstrual disorders in hospital-based cohorts [48,49]. Macroprolactin was excluded by PEG precipitation in all cases [23]. Hyperprolactinemia disrupts ovulatory function through kisspeptin-mediated inhibition of GnRH and secondary increases in adrenal androgen secretion [50,51]. For borderline-elevated prolactin values, repeat sampling is recommended before initiating treatment [23].

#### 4.1.5. Cross-Axis Endocrine Interactions and the PAInT/PMOS Construct

A key feature of the PAInT framework is that its four axes do not operate in isolation. In the present cohort, 41.8% of participants with IR also met the criteria for hyperandrogenemia, consistent with the established bidirectional relationship between hyperinsulinemia and androgen excess; elevated insulin directly stimulates ovarian theca cell androgen synthesis [6,38,39] while simultaneously suppressing hepatic SHBG production, amplifying free androgen bioavailability [34]. Conversely, androgen excess may worsen IR through adipose tissue redistribution and impairment of insulin receptor signaling [35]. The adrenal-predominant pattern in adolescents (DHEAS-driven) is mechanistically consistent with hyperinsulinemia-mediated adrenal androgen hypersecretion during the pubertal transition [15,35]. Hyperprolactinemia, present in approximately one in eight participants irrespective of age group, may independently amplify OD through kisspeptin-mediated GnRH suppression [50] and secondary adrenal androgen stimulation [51]. Thyroid dysfunction, while detected at low absolute prevalence using the primary threshold, interacts with both the insulin and androgen axes through effects on SHBG synthesis and insulin sensitivity [45,46]. Taken together, these inter-axis interactions support the PMOS construct [7], in which OD results not from a single endocrine lesion but from the co-occurrence and mutual amplification of disturbances across the four PAInT axes.

### 4.2. Clinical Integration: PAInT Framework and PMOS Context

This study’s contribution is specifically epidemiological: it demonstrates that the four endocrinopathies integrated in the PAInT framework are already detectable and clinically prevalent during adolescence, at frequencies that do not differ significantly from those observed in young adult women. This finding provides an empirical basis for structured PAInT-based endocrine evaluation in women with OD ≥ 2 years post-menarche [8,9,10,11,52], irrespective of reproductive intent. It does not, by itself, support initiating pharmacological treatment in adolescents; rather, it supports the rationale for systematic endocrine identification so that those who require treatment, guided by established clinical criteria [12], can be identified promptly. It should be acknowledged, however, that not all forms of ovulatory dysfunction are equally well characterized within the PAInT framework [53]; luteal phase deficiency, in particular, remains an entity with ongoing diagnostic heterogeneity and limited clinical consensus, and its evaluation requires a distinct methodological approach beyond the scope of the four-axis protocol applied here [54].

### 4.3. Limitations

The following limitations are explicitly acknowledged: (i) The absence of a concurrent internal control group with confirmed ovulatory function is the principal structural limitation of this study. Without a matched comparator from the same clinical setting, it is not possible to determine with certainty whether the observed endocrinopathy prevalences are specifically attributable to ovulatory dysfunction or partially reflect the background endocrine profile of symptomatic women at a specialized center. The four external reference populations (Burrows et al. [28] for IR; ENS 2016–2017 [17] for thyroid dysfunction; asymptomatic blood donors [29] for hyperprolactinemia; and population-based estimates [30] for hyperandrogenism) represent the best available validated benchmarks, yet they differ from the study cohort in ascertainment method, symptom burden, age distribution, and for the thyroid reference, diagnostic threshold. All between-cohort comparisons are therefore strictly descriptive and directional, and they cannot be interpreted as providing odds ratios or attributable fractions. A prospective case-control study recruiting ovulatory women from the same setting under identical hormonal protocols would be required to establish the endocrinopathy prevalence specifically attributable to OD. (ii) The study was not powered or designed for multivariable modelling; only unadjusted exploratory comparisons were performed, so independent effects of age and other confounders cannot be formally assessed. (iii) Immunoassay methodology: hyperandrogenemia prevalences are upper-bound estimates pending LC-MS/MS validation. (iv) Single-center design: findings may not represent the ethnic, socioeconomic, or geographic diversity of Chilean or Latin American populations. (v) Anti-Mullerian Hormone (AMH) was not included in the hormonal panel, as the study was designed to characterize the prevalence of endocrinopathies within the four-axis PAInT framework rather than to establish PMOS diagnosis. Future studies should incorporate AMH measurement to enable complete PMOS phenotyping per the current international guidelines [12]. (vi) The absence of systematic Tanner staging precludes the precise separation of physiological pubertal insulin resistance from pathological metabolic insulin resistance in adolescents.

## 5. Conclusions

Hyperandrogenemia, IR, thyroid dysfunction, and hyperprolactinemia were all detectable during adolescence in women with confirmed OD, with prevalences largely comparable to those observed in young adult women; IR was the sole endocrinopathy significantly more frequent in the adolescent stratum. The observed four-endocrinopathy co-occurrence pattern is consistent with the polyendocrine–metabolic framework formalized in the PMOS nomenclature [7] and provides an empirical basis for structured PAInT-based endocrine evaluation [8,9,10,11,23,24,25,26] in women with OD ≥2 years post-menarche, irrespective of reproductive intent. This epidemiological contribution does not, by itself, support initiating pharmacological treatment in adolescents; rather, it supports the rationale for systematic endocrine identification so that those who require intervention, guided by the established clinical criteria, can be recognized promptly. These findings are consistent with recommendations for structured endocrine evaluation in adolescents with persistent OD beyond physiological post-menarche maturation [12,52], although specific entities such as luteal phase deficiency continue to present diagnostic heterogeneity and limited consensus [54] and suggest that longitudinal studies are warranted to clarify the long-term reproductive and cardiometabolic implications of early-onset endocrine dysregulation.

## Figures and Tables

**Table 1:** Demographic and anthropometric characteristics.

Variable	Group A (*n* = 96)	Group B (*n* = 155)	*p*-Value
Age (years), median (IQR)	16 (14–17)	26 (22–30)	<0.0001 **
Weight (kg), median (IQR)	60.3 (55.1–66.4)	64.0 (57.0–70.7)	0.020 *
Height (m), mean (±SD)	1.62 (±0.057)	1.64 (±0.063)	0.079
Overweight, *n* (%)	28 (29.2%)	43 (27.7%)	0.808
Obesity, *n* (%)	8 (8.3%)	12 (7.7%)	0.866
Overweight or obesity, *n* (%)	36 (37.5%)	55 (35.5%)	0.747

* *p* < 0.05; ** *p* < 0.01.

**Table 2:** Main reason for consultation by study group.

Symptom	Group A (*n* = 96)	Group B (*n* = 155)	*p*-Value
Irregular menses	56 (58.3%)	75 (48.4%)	0.138
Acne	39 (40.6%)	29 (18.7%)	<0.001 **
Dysmenorrhea	24 (25.0%)	30 (19.4%)	0.290
Weight gain	25 (26.0%)	47 (30.3%)	0.447
Amenorrhea	19 (19.8%)	23 (14.9%)	0.307
Hirsutism	14 (14.6%)	15 (9.7%)	0.245
Pre-menstrual syndrome	1 (1.0%)	6 (3.9%)	0.183
Alopecia	1 (1.0%)	5 (3.2%)	0.268
Galactorrhea	2 (2.1%)	7 (4.5%)	0.309
Menorrhagia	2 (2.1%)	3 (1.9%)	0.709
Infertility	0 (0.0%)	25 (16.1%)	<0.001 **

More than one symptom could be recorded per participant. **: *p* < 0.01.

**Table 3:** Basic hormonal profile by study group.

Hormone/Index	Unit	Group A Median (IQR)	Group B Median (IQR)	*p*-Value
FSH	IU/L	5.11 (4.20–5.90)	6.45 (5.60–8.00)	<0.0001 **
Estradiol	pg/mL	36.18 (24.00–54.50)	55.00 (31.70–71.00)	0.005 **
TT	ng/dL	29.45 (20.00–45.00)	35.00 (22.00–50.80)	0.252
CFT	pg/mL	3.86 (2.59–6.33)	4.13 (2.40–6.50)	0.828
SHBG	nmol/L	50.91 (35.80–77.80)	58.15 (45.50–75.00)	0.110
DHEAS	μg/dL	218.00 (165.00–303.00)	191.00 (142.00–257.00)	0.022 *
A4	ng/mL	2.55 (1.63–3.20)	2.30 (1.40–3.50)	0.944
TSH	μIU/mL	1.95 (1.30–2.65)	1.77 (1.11–2.70)	0.282
PRL	ng/mL	11.00 (7.30–16.10)	11.80 (7.73–17.47)	0.576
Fasting glycemia	mg/dL	85.00 (81.00–92.00)	86.00 (81.00–90.50)	0.887
Fasting insulin	μIU/mL	10.30 (6.80–15.20)	7.35 (5.10–10.20)	0.002 **
HOMA index	—	2.16 (1.26–3.50)	1.59 (1.05–2.20)	0.003 **

*: *p* < 0.05; **: *p* < 0.01.

**Table 4:** Prevalence of endocrinopathies by study group.

Variable	Group A (*n* = 96)	Group B (*n* = 155)	*p*-Value
INSULIN RESISTANCE			
≥1 criterion positive	53 (55.2%)	64 (41.3%)	0.032 *
HOMA index > 2.09	46 (47.9%)	42 (27.1%)	0.001 **
ISI composite < 4.45	45 (46.9%)	52 (33.6%)	0.035 *
I_0_ × G_60_ > 1110	35 (36.5%)	43 (27.7%)	0.147
HYPERANDROGENEMIA			
≥1 criterion positive	48 (50.0%)	76 (49.0%)	0.882
TT > 47 ng/dL	22 (22.9%)	48 (31.0%)	0.167
CFT > 9 pg/mL	11 (11.5%)	16 (10.3%)	0.778
DHEAS > 250 μg/dL	36 (37.5%)	38 (24.5%)	0.028 *
A4 > 2.8 ng/mL	28 (29.2%)	46 (29.7%)	0.931
THYROID DYSFUNCTION			
TSH > 5.0 μIU/mL (primary threshold) (a)	2 (2.1%)	8 (5.2%)	0.226
TSH 2.5–5.0 μIU/mL (URR—exploratory) (b)	25 (26.0%)	36 (23.2%)	0.613
Any TSH ≥ 2.5 μIU/mL (exploratory) (b)	27 (28.1%)	44 (28.4%)	0.964
HYPERPROLACTINEMIA			
PRL > 25 ng/mL	13 (13.5%)	21 (13.6%)	0.999
PREMATURE OVARIAN INSUFFICIENCY			
FSH > 25 IU/L + E2 < 50 pg/mL	0 (0.0%)	2 (1.3%)	0.264

(a) Diagnostic category based on established clinical guideline thresholds; (b) exploratory analytical category. * *p* < 0.05; ** *p* < 0.01.

## Data Availability

The original contributions presented in this study are included in the article. Further inquiries can be directed to the corresponding authors.

## Abbreviations

The following abbreviations are used in this manuscript:

17-OHP	17-hydroxyprogesterone
A4	Androstenedione
ATA	American Thyroid Association
ASRM	American Society for Reproductive Medicine
BHP	Basic hormonal profile
CFT	Calculated free testosterone
DHEAS	Dehydroepiandrosterone sulfate
E2	Estradiol
ENS	Encuesta Nacional de Salud (Chilean National Health Survey)
FSH	Follicle-stimulating hormone
GnRH	Gonadotropin-releasing hormone
HOMA	Homeostatic model assessment index
HPAG	Hypothalamic–pituitary–adrenal–gonadal axis
I_0_ × G_60_	Product of fasting insulin × glucose at 60 min
IR	Insulin resistance
ISI	Insulin sensitivity index composite
LC-MS/MS	Liquid chromatography-tandem mass spectrometry
LH	Luteinizing hormone
OD	Ovulatory dysfunction
OGTT-I	Oral glucose tolerance test with insulin response
PAInT	Prolactin–Androgens–Insulin–Thyroid clinical framework
PCOS	Polycystic ovary syndrome
PMOS	Polyendocrine metabolic ovarian syndrome
POI	Premature ovarian insufficiency
PRL	Prolactin
RHRI	Reproductive Health Research Institute
SHBG	Sex hormone-binding globulin
TSH	Thyroid-stimulating hormone
TT	Total testosterone
URR	Upper reference range (TSH 2.5–5.0 μIU/mL; exploratory)
